# The small molecule AUTEN-99 (autophagy enhancer-99) prevents the progression of neurodegenerative symptoms

**DOI:** 10.1038/srep42014

**Published:** 2017-02-16

**Authors:** Tibor Kovács, Viktor Billes, Marcell Komlós, Bernadette Hotzi, Anna Manzéger, Anna Tarnóci, Diána Papp, Fanni Szikszai, Janka Szinyákovics, Ákos Rácz, Béla Noszál, Szilvia Veszelka, Fruzsina R. Walter, Mária A. Deli, Laszlo Hackler, Robert Alfoldi, Orsolya Huzian, Laszlo G. Puskas, Hanna Liliom, Krisztián Tárnok, Katalin Schlett, Adrienn Borsy, Ervin Welker, Attila L. Kovács, Zsolt Pádár, Attila Erdős, Adam Legradi, Annamaria Bjelik, Károly Gulya, Balázs Gulyás, Tibor Vellai

**Affiliations:** 1 Velgene Biotechnology Research Ltd., Szeged, H-6726, Hungary; 2 Department of Genetics, Eötvös Loránd University, Budapest, H-1117, Hungary; 3 Department of Pharmaceutical Chemistry, Semmelweis University, Budapest, H-1092, Hungary; 4 Group of Biological Barriers, Institute of Biophysics, Biological Research Centre, Hungarian Academy of Sciences, Szeged, H-6726, Hungary; 5 Avidin Ltd., Szeged, H-6726, Hungary; 6 Department of Physiology and Neurobiology, Eötvös Loránd University, Budapest, H-1117, Hungary; 7 MTA-ELTE NAP B Neuronal Cell Biology Research Group, Eötvös Loránd University, Budapest, H-1117, Hungary; 8 Institute of Enzymology, Research Centre for Natural Sciences, Budapest, H-1117, Hungary; 9 Department of Anatomy, Cell and Developmental Biology, Eötvös Loránd University, Budapest, H-1117, Hungary; 10 Department of Cell Biology and Molecular Medicine, University of Szeged, Szeged, H-6720, Hungary; 11 Karolinska Institute, Department of Clinical Neuroscience, S-171 76 Stockholm, Sweden; 12 Lee Kong Chian School of Medicine, Nanyang Technological University, 636921 Singapore; 13 Imperial College London, Department of Medicine, Division of Brain Sciences, London, SW7 2AZ, UK

## Abstract

Autophagy functions as a main route for the degradation of superfluous and damaged constituents of the cytoplasm. Defects in autophagy are implicated in the development of various age-dependent degenerative disorders such as cancer, neurodegeneration and tissue atrophy, and in accelerated aging. To promote basal levels of the process in pathological settings, we previously screened a small molecule library for novel autophagy-enhancing factors that inhibit the myotubularin-related phosphatase MTMR14/Jumpy, a negative regulator of autophagic membrane formation. Here we identify AUTEN-99 (autophagy enhancer-99), which activates autophagy in cell cultures and animal models. AUTEN-99 appears to effectively penetrate through the blood-brain barrier, and impedes the progression of neurodegenerative symptoms in *Drosophila* models of Parkinson’s and Huntington’s diseases. Furthermore, the molecule increases the survival of isolated neurons under normal and oxidative stress-induced conditions. Thus, AUTEN-99 serves as a potent neuroprotective drug candidate for preventing and treating diverse neurodegenerative pathologies, and may promote healthy aging.

Various neurodegenerative conditions are associated with the progressive accumulation of damaged, dysfunctional proteins that can act as cellular toxins^1^,^2^,^3^,^4^,^5^. For example, Alzheimer’s disease (AD) is associated with the accumulation of β-amyloid or hyperphosphorylated tau proteins^6^. Parkinson’s disease (PD) is triggered by deposits of mutant α-synuclein or Parkin proteins in dopaminergic neurons^7^. Huntington’s disease (HD) results from the collection of a mutant Huntingtin protein (HTT) containing a long (over 39) polyglutamine repeat in the N terminus^8^. The progressive accumulation of such toxic proteins can lead to massive levels of neuronal cell death, which underlie the manifestation of neurodegenerative symptoms.

The effective elimination of harmful proteins and other damaged constituents from the cytoplasm is essential to maintain neuronal homeostasis and tissue functioning, and is primarily achieved by autophagy, a main form of cellular self-degradation^1^,^2^,^3^,^4^,^5^,^9^. In addition, accumulating evidence reveals that several aggregation-prone proteins implicated in neuronal degeneration normally play a role in the mechanism of autophagy. For example, Parkin is required for the targeted elimination of damaged mitochondria, the process called mitophagy^10^,^11^ while HTT functions as a scaffold for selective autophagy^12^,^13^. During autophagy, parts of the cytoplasm are delivered into the lysosomal system that contains acidic hydrolases including proteases, nucleases, lipases and glycosidases. Depending on the mechanism of delivery, three major forms of autophagy can be distinguished: microautophagy (the lysosomal membrane directly internalizes cytoplasmic materials through invagination), chaperone-mediated autophagy (specific chaperones bind to certain cytoplasmic proteins and transport them into the lysosomal lumen through the membrane protein LAMP2A) and macroautophagy^4^,^14^. Macroautophagy (hereafter referred to as autophagy) is initiated by the formation of a double membrane structure, which grows around the cytoplasmic material destined for degradation^9^,^15^. When the sequestration of cargo becomes completed, a double membrane-bound vesicle called autophagosome is formed. The autophagosome then fuses with a lysosome to form an autolysosome in which the molecular degradation occurs. Generation and maturation of the autophagosomal membrane require several evolutionarily conserved autophagy-related proteins (ATG)^1^,^2^,^3^,^4^,^9^,^15^. These factors form distinct protein complexes to execute the autophagic process. One of them is the class III PtdIns3K (phosphatidylinositol 3-kinase) complex that involves PtdIns3K, the ortholog of yeast Vps34 (phosphatidylinositol 3-kinase Vps34)^16^. PtdIns3K converts PtdIns (phosphatidylinositol) into PtdIns3P (phosphatidylinositol 3-phosphate), which constitutes an essential component of the autophagosomal and endosomal membranes (Fig. 1a)^16^. The generation of PtdIns3P from PtdIns is a reversible process; myotubularin-related phosphatases (MTMRs), including MTMR14 also called Jumpy, antagonizes PtdIns3K to inhibit the autophagic process (Fig. 1a)^17^,^18^,^19^. MTMR14 hence functions to inhibit injurious hyperactivation of autophagy which can lead to the loss of the affected cell^20^,^21^,^22^.

Defects in the autophagic process are frequently associated with the incidence of various neurodegenerative diseases, many of which remain fatal^1^,^2^,^3^,^23^. In parallel, an age-dependent decline in the capacity of autophagy has been observed in the nervous system of certain organisms such as the fruit fly *Drosophila melanogaster*^24^. These observations raise the possibility that promoting basal levels of autophagy at advanced ages or activating the process in neurodegenerative pathological conditions may help prevent or alleviate such disorders in humans. Indeed, significant efforts have been expended in the last decade to isolate autophagy-inducing drugs and use them therapeutically (see refs 25, 26, 27, 28, 29, 30 as examples). However, the majority of autophagy-promoting factors identified so far have been revealed to exert undesired side effects. For example, rapamycin, a frequently used antiproliferative drug, is known to act as a potent inducer of autophagy by blocking MTOR (mechanistic target of rapamycin) kinase, an upstream inhibitor of the autophagic process^31^. MTOR is a multifunctional protein that regulates several fundamental processes beyond autophagy such as translation, ribosome biogenesis, development and aging^32^,^33^. Thus, a need for identifying novel autophagy inducers with no side effects remains at the core of current pharmacological research.

## Results

### AUTEN-99 induces autophagic flux and promotes cell survival in mammalian cell cultures

We previously screened a small molecule library for compounds that inhibit human MTMR14, a negative regulator of the autophagic process^34^. The first molecule we characterized from the candidates is AUTEN-67 (autophagy enhancer-67). This agent is capable of delaying the onset and reducing the severity of pathological features observed in a mouse and a *Drosophila* model of Alzheimer’s and Huntington’s disease, respectively^34^,^35^. Based on cell culture experiments, AUTEN-67 exerts a potent neuroprotective effect^34^.

**Figure 1:**
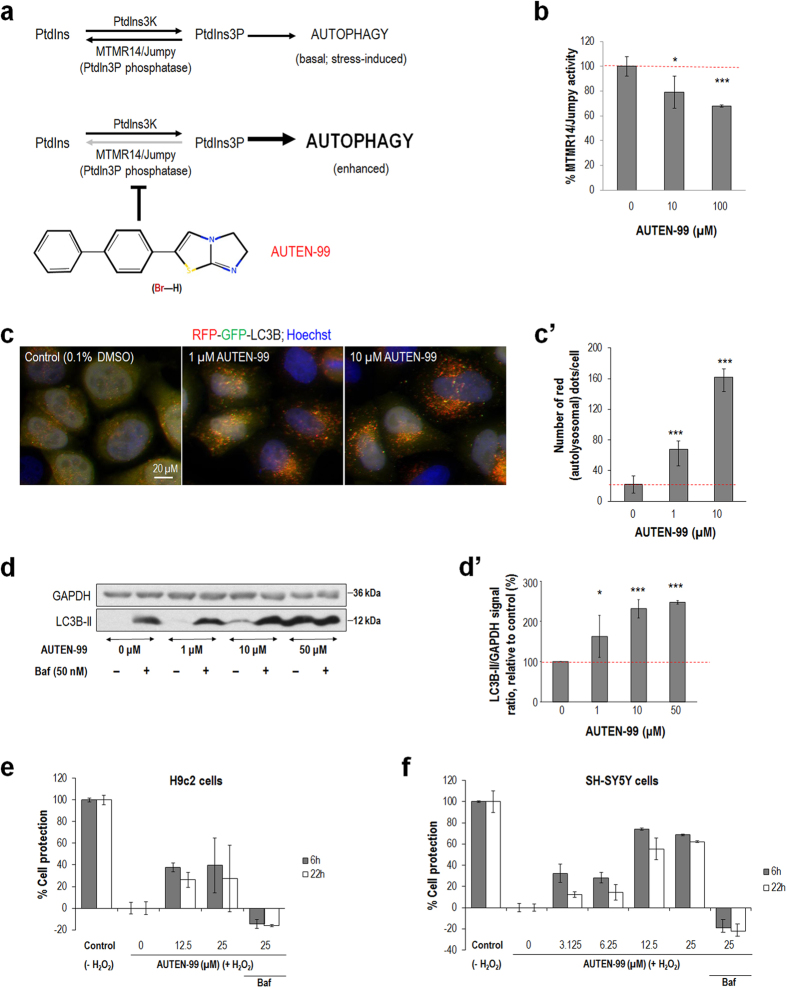
AUTEN-99 enhances autophagic flux in HeLa cells and promotes the survival of cultured mammalian cells. (**a**) A mechanistic model by which AUTEN-99 induces autophagy. AUTEN-99 impedes the human myotubularin-related phosphatase MTMR14/Jumpy, which antagonizes PtdIns3K (the human ortholog of yeast Vps34) required for generating the membrane component PtdIns3P. PtdIns: phosphatidylinositol; PtdIns3P: phosphatidylinositol 3-phosphate; PtdIns3K: phosphatidylinositol 3-kinase; MTMR: myotubularin-related phosphatase. The chemical structure of AUTEN-99 is shown (**b**) AUTEN-99 inhibits the phosphatase activity of MTMR14 in a concentration dependent manner. The red dashed line indicates average MTMR14/jumpy activity in the absence of AUTEN-99. (**c**) AUTEN-99 enhances autophagic flux in HeLa cells transgenic for the autophagy marker RFP-GFP-LC3B. Yellow dots correspond to autophagosomal, while red foci label autolysosomal structures. Autophagic structures in control (0.1% DMSO, left panel) and AUTEN-99-treated HeLa cells (middle and right panels). (c’) Quantification of autolysosomal structures (red dots) in control versus AUTEN-99-treated cells. The red dashed line indicates the average number of red foci in untreated HeLa cells. (**d**) Western blot showing that AUTEN-99 treatment elevates levels of LC3B-II, a membrane-conjugated form of LC3B, in HeLa cells in a concentration-dependent manner. GAPDH (glyceraldehyde 3-phosphate dehydrogenase) serves as an internal control. (d’) Quantification of relative LC3B-II signals upon AUTEN-99 treatment in Baf-treated cells, as seen on western blots. The red dashed line indicates average LC3B-II levels in untreated cells. Baf: Bafilomycin A1 (a potent inhibitor of autophagy). (**e**,**f**) AUTEN-99 increases the survival of H9c2 rat embryonal cardiac muscle cells (**e**) and SH-SY5Y human neuroblastoma cells (**f**) exposed to oxidative stress (H_2_O_2_ treatment). Control: cells were not exposed to H_2_O_2_ (− H_2_O_2_). The duration (in hours) of the treatment is indicated as white and gray columns. Inhibition of autophagy by Bafilomycin A1 markedly suppresses the positive effect of AUTEN-99 on cell survival. Baf: Bafilomycin A1. In panels **b**, c’, d’, **e** and **f**, bars represent mean ± S.D., *P < 0.05; **P < 0.01; ***P < 0.001; two-sample Student’s *t*-test.

Another promising candidate we obtained from the screen is T0512–8758 (2-(4-Phenylphenyl)-5,6-Dihydroimidazo[2,1-B][1,3]Thiazole), which we called AUTEN-99 (autophagy enhancer-99) (Fig. 1a). AUTEN-99 was able to decrease the phosphatase activity of MTMR14 in a concentration-dependent manner (Fig. 1b). Since MTMR14 interferes with autophagy^18^,^19^, we next monitored the effect of AUTEN-99 on autophagic activity in HeLa cells transgenic for a dually labeled key autophagy protein, LC3B (microtubule-associated protein 1 light chain 3 beta), the ortholog of yeast Atg8, that is conjugated to the forming autophagosomal membrane)^9^,^15^. The RFP-GFP-LC3B (red fluorescent protein and green fluorescent protein) reporter used in this study labels autophagosomes as yellow foci (from the merge of red and green fluorescent signals) and autolysosomes as red dots (as GFP is unstable in the acidic lumen of lysosomes). We found that AUTEN-99 markedly increases the number of autolysosomal structures in a concentration-dependent manner (Fig. 1c,c’). Consonantly, increasing AUTEN-99 concentration significantly enhanced levels of LC3B-II protein (the membrane conjugated form of LC3B) in HeLa cells (Fig. 1d,d’). These data indicate that AUTEN-99 elevates autophagic flux rather than merely strengthening the formation of autophagosomes without autolysosomal degradation in cultured human cells.

As autophagy supports cellular homeostasis and functioning, we assessed whether AUTEN-99 protects cultured mammalian cells from undergoing oxidative stress-induced death. According to our results, H9c2 rat embryonal cardiac muscle and SH-SY5Y human neuroblastoma cells exposed to H_2_O_2_ (an oxidative stress factor) survive significantly longer with the drug applied at 1–25 μM concentrations, as compared with untreated controls (Fig. 1e,f). Inhibiting autophagy by Bafilomycin A1, which prevents lysosome acidification and autophagosome-lysosome fusion, suppressed the enhanced survival of AUTEN-99-treated cells under condition of oxidative stress (Fig. 1e,f). Thus, AUTEN-99 exhibits significant cell (neuro-) protective effects via inducing the autophagic process.

### AUTEN-99 increases the number of autophagic structures in mice and *Drosophila*, and effectively penetrates through a blood-brain barrier model

**Figure 2:**
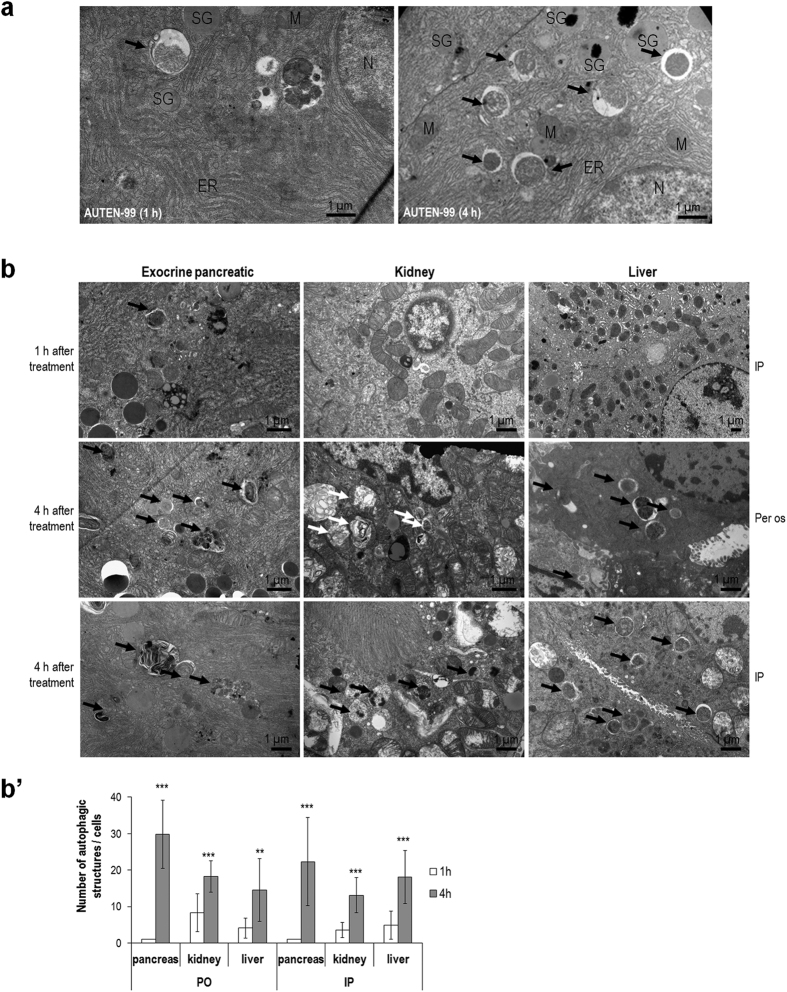
AUTEN-99 increases the number autophagic structures in mice. (**a**) Left panel: a representative transmission electron microscopy (TEM) picture showing the ultrastructure of an exocrine pancreatic cell from a mouse treated with AUTEN-99 for 1 hour only. The arrow points to an autophagic structure. Right panel: TEM image showing autophagic structures (arrows) in an exocrine pancreatic cell from a mouse treated with AUTEN-99 for 4 hours. ER: endoplasmic reticulum; M: mitochondrion; N: nucleus; SG: secretory granule. (**b**) TEM pictures showing the ultrastructure of pancreatic, liver and kidney cells from AUTEN-99-treated mice. Samples were collected 1 and 4 hours after the treatment. The administered dose for AUTEN-99 was nearly 390 μM. Arrows point to autophagic structures. IP, intraperitoneal; Per os, oral administration. (b’) Quantification of autophagic structures in TEM samples. Bars represent mean ± S.D., **p < 0.01, ***p < 0.001; paired Student’s *t*-test.

Using oral and intraperitoneal administrations, we next treated mice with AUTEN-99, and examined the formation of autophagic structures by electron microscopy in different tissues including pancreas, kidney, liver and brain (Fig. 2, Fig. S1). After 1 hour of treatment, autophagosomes and autolysosomes were either not or hardly detectable in exocrine pancreatic, kidney and liver cells (left panel in Fig. 2a, upper panels in Fig. 2b). However, 4 hours of treatment led to a massive increase in the number of autophagic structures, including late autolysosomes, in these tissue samples (right panel in Fig. 2a, middle and bottom panels in Fig. 2b). Quantification of autophagosomes and autolysosomes in treated samples clearly revealed that administration of AUTEN-99 markedly increases autophagic activity in mice (Fig. 2c). The abundant presence of late autolysosomes was indicative for an increased flux of the process. Taken together, we conclude that AUTEN-99 significantly induces autophagy in HeLa cells (Fig. 1c,d’), promotes the survival of cultured rat and human cells under oxidative stress conditions (Fig. 1g,h), and can also massively stimulate autophagic degradation in mice (Fig. 2).

**Figure 3:**
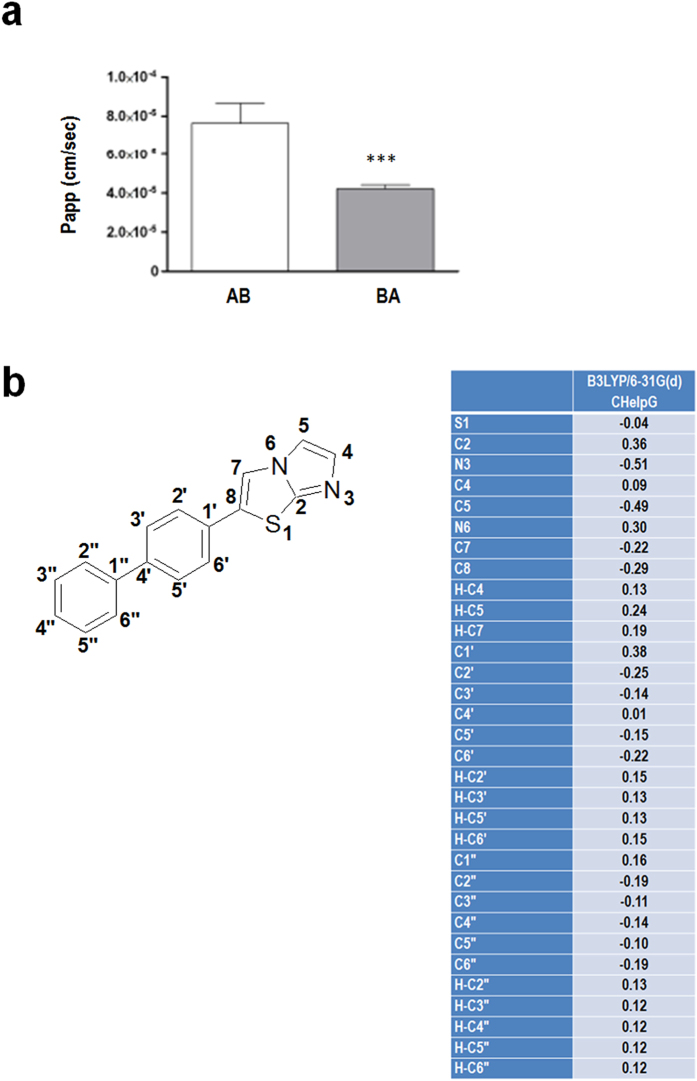
AUTEN-99 effectively crosses a blood-brain barrier culture model. (**a**) Permeability of AUTEN-99 across a blood-brain model in AB (from blood to brain) and BA (from brain to blood) directions (n = 3). P_app_ [apparent permeability coefficient (given in 10^−5^ cm/s)] shows the speed of penetration of a given material across a barrier. Permeability coefficient of AUTEN-99 is particularly high in AB direction, indicating a fast penetration across the cell layers of the model. The amount of AUTEN-99 which crossed the barrier model in AB direction was 36.24% of the total added material, while it was 13.23% in BA direction. Both values represent a very significant penetration of the molecule. Bars represent mean ± S.D., ***p < 0.001, two-sample Student’s *t*-test. (**b**) Quantum chemical calculations of AUTEN-99. Left panel: the numbering scheme of AUTEN-99. Right panel: the calculated partial charges of atoms in AUTEN-99. Significantly negative partial charge is assigned to the N3 nitrogen and its lone electron pair. Therefore, it can be protonated and is a possible hydrogen bond acceptor site. Charge distribution on carbons of the biphenyl moiety is balanced and symmetrical, which provides the molecule with high level of lipophilicity, and poor water-solubility, especially in basic media. Its lipophilicity in terms of logP value was also calculated, using the SYBYL software package (see the Materials and Methods), and it has been found to be 3.85 (with the treatment of all hydrogens, polar proximity via bond), a high level of lipophilicity.

Using a well characterized blood-brain barrier culture model^36^,^37^, we demonstrated an effective penetration of AUTEN-99 in blood to brain direction (Fig. 3a) which is comparable to the permeability coefficients of passive lipophilic reference compounds like caffein^36^,^37^. The permeability coefficient of fluorescein, a hydrophilic reference molecule was 1.57 ± 0.32 × 10^−6^ cm/s, forty times lower than that of AUTEN-99, indicating the appropriate tightness of the model. The brain-to-blood/blood-to-brain ratio of AUTEN-99 permeability was 0.55 (Fig. 3a), therefore we cannot exclude the participation of an active transport mechanism at the level of the blood-brain barrier. Quantum chemical calculations further showed that charge distribution on the carbons of the biphenyl moiety is balanced and symmetrical, which provides AUTEN-99 with high level of lipophilicity (Fig. 3b).

**Figure 4:**
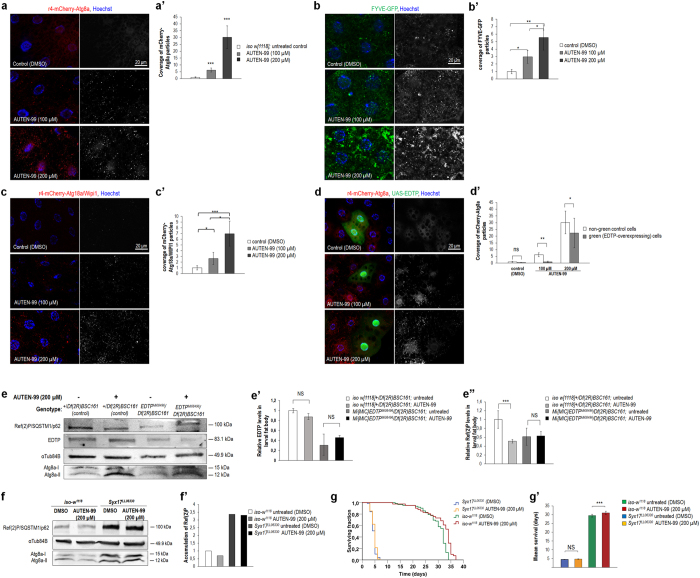
AUTEN-99 induces autophagy in the *Drosophila* larval fat body through inhibiting the MTMR14/Jumpy-like phosphatase EDTP. (**a**) AUTEN-99 treatment increases the number of mCherry-Atg8a-labeled autophagic structures in *Drosophila* larval fat body cells in a concentration-dependent manner. (a’) Quantification of Atg8a-positive structures in untreated versus AUTEN-99-treated fat body cells. (**b**) AUTEN-99 increases the number of FYVE-GFP-positive structures in a concentration-dependent manner. FYVE zinc finger domains bind PtdIns3P connected to both autophagic and endosomal functions. (b’) Quantification of FYVE-positive structures in untreated versus AUTEN-99-treated fat body cells. (**c**) AUTEN-99 treatment elevates the amount of Atg18a/WIPI1-positive autophagic structures in a concentration-dependent manner. Atg18a/WIPI1 binds PtdIns3P connected to the autophagic pathway exclusively. (c’) Quantification of Atg18a/WIPI1-labeled structures in untreated versus AUTEN-99-treated samples. (**d**) AUTEN-99 induces autophagy in larval fat body cells through interfering with EDTP, the *Drosophila* ortholog of human MTMR14/Jumpy. Fat body. (d’) Quantification of mCherry-Atg8a-labeled autophagic structures in normal and *EDTP*-overexpressing fat body cells. Hoechst staining indicates nuclei. In panels a’ to d’, bars represent mean ± S.D., *P < 0.05; **P < 0.01; ***P < 0.001; NS: not significant; two-sample Student’s *t*-test. (**e**) Representative Western blot showing relative Ref(2)P/SQSTM1/p62, EDTP, Atg8a-I and Atg8a-II levels. α-Tubulin84B serves as an internal control. (e’) Quantification of relative EDTP levels. (e”) Quantification of Ref(2)P/SQSTM1/p62 band intensities. In the EDTP-deficient genetic background (*EDTP*^*M08496*^), AUTEN-99 cannot further reduce Ref(2)P/SQSTM1/p62 levels, as compared with untreated control. *Df(2 R)BSC161* refers to a large deletion which overlaps the genomic region of *EDTP.* Blots were performed in triplicates. Bars represent mean ± S.D., **: P < 0.01; ***P < 0.001; NS: not significant; two-sample Student’s *t*-test. (**f**) AUTEN-99 cannot reduce Ref(2)P/SQSTM1/p62 levels in the autophagy deficient *Syx17*^*LL06330*^mutant background. (f’) Quantification of Ref(2)P/SQSTM1/p62 levels seen on panel **f**. (**g**) AUTEN-99 extends life span in control (*w*^*1118*^) *Drosophila* but not in *Syx17*^*LL06330*^mutant animals defective for autophagy. Mantel-Cox log rank test, Kaplan-Meier survival curves. (g’) Mean survival rate of animals shown on panel **g**. Bars represent mean ± S.E.M., ***P < 0.001; NS: not significant; Mann-Whitney U-test.

Mammalian MTMR14 proteins share highly conserved protein domains with their single *Drosophila melanogaster* ortholog, EDTP (egg-derived tyrosine phosphatase)^34^. This sequence conservation prompted us to investigate whether AUTEN-99 could modulate autophagic activity in the fat body of the fruit fly larva, a tractable genetic model for studying developmental and stress-induced autophagy^38^. Using an mCherry-Atg8a reporter (Atg8a is the *Drosophila* ortholog of human LC3B/yeast Atg8 autophagy proteins), we found that AUTEN-99 intensely elevates the amount of Atg8a-postive autophagic structures in fat body cells in a concentration-dependent manner (Fig. 4a,a’). Furthermore, a reporter labeling FYVE protein domains that bind PtdIns3P connected to both endocytic and autophagic pathways revealed a massive increase in PtdIns3K activity in response to AUTEN-99 treatment (Fig. 4b,b’). We also monitored the amount of Atg18a/WIPI1 that binds PtdIns3P functioning in the autophagic pathway only, and found a significant, concentration-dependent enlargement upon adding the compound (Fig. 4c,c’). These results convincingly demonstrate that AUTEN-99 activates the autophagic process at the level or upstream of PtdIns3K. Fat body cells clonally overexpressing EDTP (green cells in Fig. 4d), however, failed to accumulate autophagosomes and autolysosomes in the presence of 100 μM AUTEN-99 (Fig. 4d,d’). This suggests that AUTEN-99 activates autophagy through inhibiting EDTP (i.e., at the level of PtdIns3K).

SQSTM1/p62 (Sequestosome-1) protein is known to serve as a substrate for autophagic degradation, thereby its levels inversely correlate with the activity of the process^39^. The level of Ref(2)P (refractory to sigma P), the sole *Drosophila* ortholog of mammalian SQSTM1/p62, was decreased in fat body samples from flies treated with AUTEN-99 (Fig. 4e,e’), serving as additional evidence that the compound induces autophagic degradation. In contrast, AUTEN-99 could not lower Ref(2)P levels further in an EDTP-defective genetic background, as compared with untreated *EDTP*^*MI08496*^ mutant samples (Fig. 4e,e’). Thus, in this organism AUTEN-99 appears to affect the autophagic process through inhibiting EDTP.

To confirm that AUTEN-99 lowers Ref(2)P/SQSTM1/p62 levels through enhancing autophagy, the amount of protein was assayed in wild-type versus autophagy-defective genetic backgrounds. Indeed, AUTEN-99 could not reduce Ref(2)P/SQSTM1/p62 levels in *Syx17*^*LL06330*^ (*Syntaxin-17*) mutant animals that are deficient in SNARE complex functions required for autophagosome-lysosome fusion^40^ (Fig. 4f,f’). Consistent with these results, AUTEN-99 extended life span in wild-type, but not in *Syx17*^*LL06330*^ mutant animals (Fig. 4g,g’). We conclude that the compound has an anti-aging effect by inducing autophagic activity.

### AUTEN-99 hampers the progression of neurodegenerative symptoms in *Drosophila* models of Parkinson’s disease

**Figure 5:**
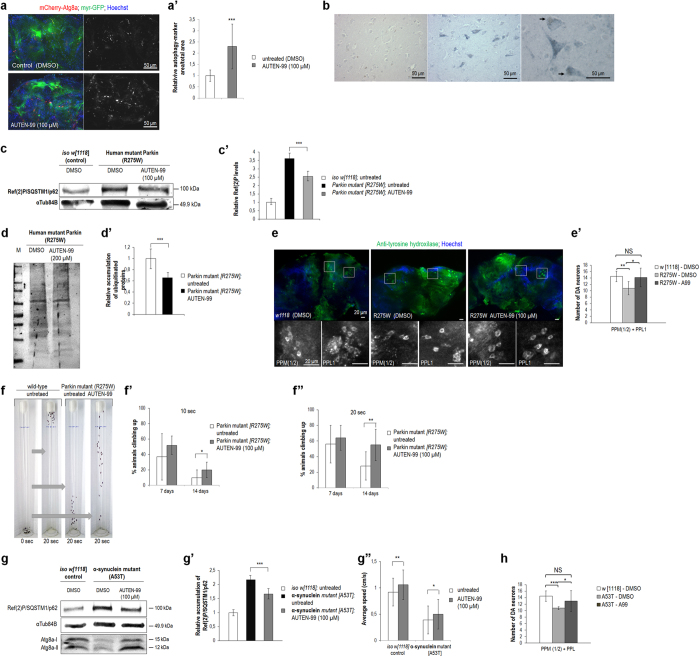
AUTEN-99 prevents the progression of neurodegenerative symptoms in *Drosophila* models of Parkinson’s disease. (**a**) AUTEN-99 increases the amount of autophagic structures in dopaminergic and serotoninergic neurons. Hoechst staining indicates nuclei. (a’) Quantification of mCherry-Atg8a-labeled autophagic structures in untreated vs. AUTEN-99-treated neurons. (**b**) Localization of MTMR14/hJumpy in neurons of the gyrus temporalis superior of a non-demented 82-year old female. Left: sample stained without antibody; middle: sample stained with an MTMR14-specific antibody; right: lipofuscin droplets (arrows). (**c**) AUTEN-99 decreases Ref(2)P/SQSTM1/p62 levels in the head of flies expressing human mutant Parkin (R275W). (c’) Quantification of Ref(2)P/SQSTM1/p62 levels in untreated versus AUTEN-99-treated animals expressing Parkin R275W. (**d**) Western blot demonstrating that AUTEN-99 decreases ubiquitinated protein levels in the brain of flies expressing Parkin R275W. (d’) Quantification of ubiquitinated proteins in untreated vs. AUTEN-99-treated animals expressing Parkin R275W. (**e**) AUTEN-99 inhibits the loss of dopaminergic and serotoninergic neurons in flies expressing Parkin R275W. Brain samples from 22 day-old flies were stained with anti-tyrosine hydroxylase specific for dopaminergic neurons. PPM: posterior protocerebrum medialis; PPL: posterior protocerebrum lateralis. (e’) Quantification of dopaminergic neurons. AUTEN-99 restores the number of dopaminergic neurons to normal levels in animals expressing Parkin R275W. (**f**) Climbing assay with *Drosophila* adults expressing Parkin R275W. (f’ and f”) Quantification of climbing ability in animals treated with DMSO or AUTEN-99. Adult animals at ages of 7 and 14 days were scored for 10 (f’) and 20 sec (f”). (**g**) AUTEN-99 decreases Ref(2)P/SQSTM1/p62 levels in the head of flies transgenic for a human mutant α-synuclein (A53T). (g’) Quantification of Ref(2)P/SQSTM1/p62 levels in flies expressing α-synuclein A53T. (g”) AUTEN-99 improves the speed at which animals expressing α-synuclein A53T climbs in a glass vial. (**h**) AUTEN-99 prevents the loss of dopaminergic neurons in animals expressing α-synuclein A53T. In panels **a’** to g’, g” and **h**, bars represent mean ± S.D., *P < 0.05; **P < 0.01, ***P < 0.001; Mann-Whitney U-test, or *t*-test for unequal variances, or two-sample Student’s *t*-test. In panels **c** to **d**, animals were tested at the adult age of 21 days. In panels **c** and **g**, α-Tubulin84B represents an internal control. In panels c’ to e’ and g’, blots were performed in triplicates.

We next examined autophagy-enhancing effects of AUTEN-99 in the *Drosophila* brain. The small molecule increased the number of autophagic structures in dopaminergic and serotonergic neurons labeled by a myr-GFP reporter (driven by *Ddc-Gal4*), which are implicated in the control of diverse biological functions including behavior, locomotion and perception (Fig. 5a,a’). We also demonstrated the neuronal accumulation of MTMR14/hJumpy in human brain samples (Fig. 5b), providing a rationale for the approach. A *Drosophila* model of PD is represented by flies expressing the human mutant Parkin protein that contains the amino acid change R275W^41^ (normal Parkin is recruited to dysfunctional mitochondria to mediate their selective autophagic elimination called mitophagy^10^,^11^). AUTEN-99 also significantly decreased Ref(2)P/SQSTM1/p62 levels (i.e., activated autophagic breakdown) in the head of this *Drosophila* PD model at 21 days of adult age (Fig. 5c,c’). In good accordance with these results, ubiquitinated proteins accumulated at lower levels in head samples from 21 day-old adults treated with AUTEN-99 than in untreated, age-matched samples (Fig. 5d,d’). As ubiqutination frequently marks harmful proteins prior to degradation, we conclude that AUTEN-99 improves general protein quality, thereby strengthening cellular homeostasis in neurons of old animals.

*Drosophila* overexpressing human mutant Parkin (R275W) was previously shown to display dopaminergic neuron (DN) degeneration and mitochondrial abnormalities^41^. These data prompted us to investigate whether AUTEN-99 can inhibit the loss of DNs in this genetic background. By determining the amount of DNs in the PPL1 (posterior protocerebrum lateralis) and PPM1/2 (posterior protocerebrum medialis) brain clusters, we observed that the compound restores the number of these cells to nearly normal levels found in age-matched controls (Fig. 5e,e’). This fly model of PD also exhibits an impaired climbing ability; while wild-type animals rapidly climb up on the wall of glass vials (this phenomenon is termed as negative geotaxis), R275W transgenic animals are unable to perform this behavioral pattern and largely remain at the bottom of tubes (Fig. 5f)^41^. AUTEN-99 treatment significantly improved locomotion in *Drosophila* transgenic for Parkin R275W mutant (Fig. 5f’,f”). The majority of treated animals, especially at age 14 days of adulthood, were able to get on the wall of vials within 20 seconds.

Another *Drosophila* model of PD expresses β-sheets of human α-synuclein that result from an amino acid change, A53T^42^. We found that the level of Ref(2)P/SQSTM1/p62 is much higher in the head of A53T mutant, 21 day-old flies than in age-matched control animals, and that this amount is significantly decreased in response to AUTEN-99 treatment (Fig. 5g,g’). In good agreement with these results, AUTEN-99 largely increased the accumulation of Atg8a-II in animals expressing the toxic α-synuclein (Fig. 5g). This indicates that AUTEN-99 improves autophagic activity in the A53T mutant *Drosophila* PD model too. Furthermore, the speed at which flies overexpressing the mutant α-synuclein climb on the wall of test vials was increased by adding AUTEN-99 to the growth medium (Fig. 5g”). The compound also restored the number of DNs to nearly normal levels in A53T mutant genetic background (Fig. 5h). Based on these data we assume that the onset and progression of neurodegenerative symptoms generated by the expression of human mutant Parkin and α-synuclein proteins can be delayed or hampered by adding AUTEN-99.

### AUTEN-99 reduces the severity of neurodegenerative symptoms in a *Drosophila* model of Huntington’s disease

**Figure 6:**
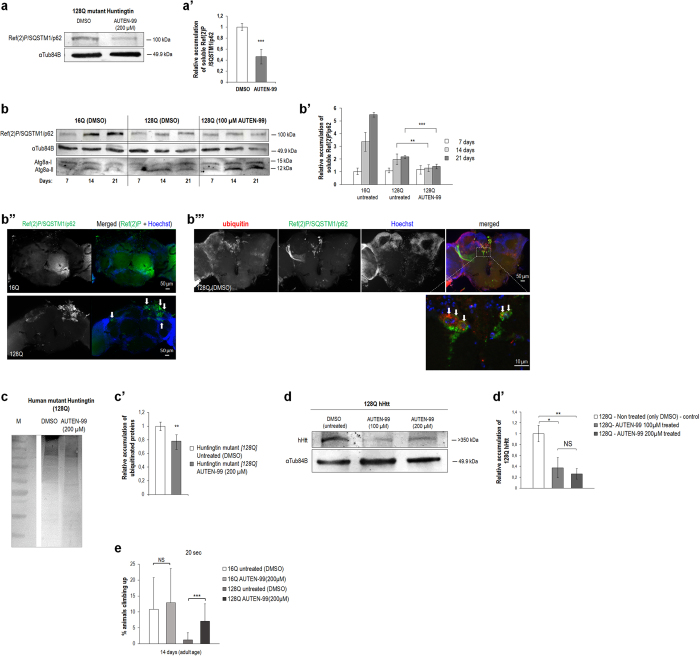
AUTEN-99 impedes the progression of neurodegenerative symptoms in a *Drosophila* model of Huntington’s disease. (**a**) Western blot showing that AUTEN-99 decreases soluble Ref(2)P/SQSTM1/p62 protein levels in head samples from a *Drosophila* model of HD (animals express the 128Q mutant form of human Huntingtin protein; 128Q-hHTT). (a’) Quantification of relative soluble Ref(2)P/SQSTM1/p62 levels (based on three independent western blots). (**b**) A representative Western blot showing an age-dependent accumulation of soluble Ref(2)P/SQSTM1/p62 proteins in the brain of flies expressing the normal (16Q) or mutant (128Q) form of hHTT. DMSO: untreated controls. AUTEN-99 inhibits an age-dependent increase in Ref(2)P/SQSTM1/p62 levels in mutant (128Q) samples. Atg8a-I (soluble) and –II (membrane conjugated) forms are also indicated. (b’) Quantification of Ref(2)P/SQSTM1/p62 levels (experiments were performed in triplicates). (b”) Fluorescent microscopy showing the aggregation of Ref(2)P/SQSTM1/p62 proteins (green) in the head sample of a fly transgenic for 128Q-hHTT. Arrows point to green foci. In the control sample (16Q), Ref(2)P/SQSTM1/p62 appears to be evenly distributed. (b”’) Ubiquitinated proteins (red) are colocalized with Ref(2)P/SQSTM1/p62 aggregates (green). Hoechst staining (blue) indicates nuclei. Arrows indicate ubiquitinated/Ref(2)P-positive aggregates. (**c**) Western blot showing levels of ubiquitinated proteins in head samples of flies expressing 128Q-hHTT. (c’) Quantification of relative ubiqutinated protein levels by analyzing three independent western blots. M: molecular weight marker. (**d**) Western blot showing that AUTEN-99 markedly decreases the levels of mutant hHTT (128Q), as compared to untreated control. (d’) Quantification of 128Q-hHTT levels in untreated (DMSO) versus AUTEN-99 treated samples. (**e**) AUTEN-99 treatment restores the climbing ability of flies expressing mutant hHTT (128Q) to nearly normal levels. 16Q refers to the normal hHTT. In panels **a, b** and **d**, αTub84B was used as an internal control. In panels a’, b’, c’, d’ and e’, bars represent mean ± S.D., *P < 0.05; **P < 0.01; ***P < 0.001; NS: not significant; Mann-Whitney U-test. The expression of normal and mutant form of hHTT was driven by *Appl-Gal4*.

*Drosophila* strain transgenic for a human mutant HTT protein represents a genetic model for HD; the toxic protein contains a 128-long polyglutamine repeat (128Q-hHTT)^42^. When compared with untreated animals, administration of AUTEN-99 strongly elevated autophagic activity in these transgenic flies, as indicated by reduced Ref(2)P/SQSTM1/p62 levels (Fig. 6a,a’). An age-dependent accumulation of Ref(2)P/SQSTM1/p62 was evident in head samples of control (expressing nontoxic – 16Q – hHTT proteins or untreated 128Q-hHTT mutants) flies which reflects a natural, progressive decline in the capacity of the autophagic process (Fig. 6b,b’). Treatment with AUTEN-99 was sufficient to maintain relatively low levels of Ref(2)P/SQSTM1/p62 even in old (at day 21) 128Q-hHTT animals (Fig. 6b,b’). Consistently with these data, Atg8a-II levels became significantly increased over the adulthood in AUTEN-99-treated animals (Fig. 6b). Interestingly, Ref(2)P/SQSTM1/p62 levels were markedly lowered in 128Q-hHTT brain samples than in (control) 16Q-hHTT ones in the absence of treatment. We suggest that autophagy is induced in the presence of the toxic hHTT protein as a result of compensatory mechanism. Alternatively, the majority of 128Q-hHTT may accumulate into insoluble protein aggregates that are not detectable by the Western-bolt analysis protocol we used in this study. To address this issue we stained brain samples with a Ref(2)P-specific antibody, and found that the protein accumulates in large foci corresponding to protein aggregates (Fig. 6b”). Based on these data we can conclude that a significant portion of Ref(2)P/SQSTM1/p62 presents in an insoluble form in animals expressing 128Q-hHTT^35^. Nevertheless, the relative amount of Ref(2)P/SQSTM1/p62 was significantly decreased in 128Q-hHTT animals when they were treated with AUTEN-99 (Fig. 6a,b’). The immunohistochemical analysis of Ref(2)P/SQSTM1/p62 also revealed that in 128Q-hHTT brain samples this autophagic substrate largely colocalizes with ubiquitin-positive protein aggregates, suggesting its receptor-like role in eliminating damaged proteins via selective autophagy (Fig. 6b”’). Consistent with these data, head samples from 21 day-old adult *Drosophila* of this HD model exhibited reduced levels of ubiquitinated proteins when the animals were treated with AUTEN-99 (Fig. 6c,c’). More significantly, AUTEN-99 markedly decreased the amount of toxic 128Q-hHTT proteins, as compared with untreated animals (Fig. 6d,d’). Administration of the drug also restored the climbing ability of 128Q-hHTT mutant adults to levels found in control (16Q-hHTT) animals (Fig. 6e). Thus, AUTEN-99 effectively impedes certain features of neuronal demise in a fly model of HD, including the progressive accumulation of damaged and toxic proteins in the brain and the inability of animals to climb up to the top of test vials.

### AUTEN-99 promotes the survival of isolated neurons under oxidative stress-induced conditions

**Figure 7:**
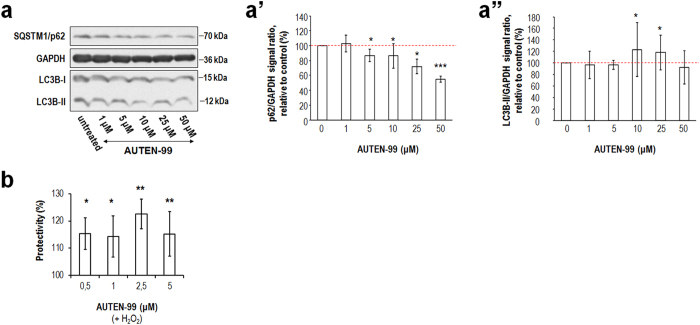
AUTEN-99 promotes the survival of isolated neurons under oxidative stress-induced conditions. (**a**) Western blot showing SQSTM1/p62 levels in isolated neurons from mice. GAPDH serves as an internal control. Soluble (I) and membrane-conjugated (II) LC3B forms are also shown. (a’) AUTEN-99 decreases the relative amount of SQSTM1/p62 proteins in a concentration-dependent manner. (a”) Relative LC3B-II levels are increased in response to AUTEN-99 treatment. (**b**) AUTEN-99 increases the survival (% protectivity) of isolated neurons exposed to oxidative stress (triggered by 50 μM H_2_O_2_ treatment). In panels a’ to **b**, bars represent ± S.E.M., *P < 0.05; **P < 0.01; ***P < 0.001; two-sample Student’s *t*-test. In panels a’ and a”, red dashed lines represent untreated (control) values.

Finally, we tested autophagic activity in untreated versus AUTEN-99-treated in isolated mouse neuronal cells. According to our results, AUTEN-99 treatment lowered SQSTM1/p62 levels in a concentration-dependent manner (Fig. 7a,a’). Concordantly, relative LC3B/Atg8-II levels were slightly elevated by 10 to 25 μM of AUTEN-99 (Fig. 7a”). These results indicate that AUTEN-99 promotes autophagic activity in isolated primary neurons in a concentration dependent manner. Moreover, AUTEN-99 triggered potent neuroprotective effects when it was applied during oxidative stress evoked by 50 μM H_2_O_2_ treatment (Fig. 7b). In summary, AUTEN-99 exerts potent neuroprotective effects in isolated neurons exposed to oxidative stress.

## Discussion

In this study, we reported the identification and initial characterization of a small molecule, AUTEN-99 (2-(4-Phenylphenyl)-5,6-Dihydroimidazo[2,1-B][1,3]Thiazole), with a potent autophagy-enhancing capacity. AUTEN-99 affected the core autophagic process via inhibiting MTMR14/hJumpy, a negative regulator of PtdIns3K critical for autophagosomal membrane formation (Figs 1a,b and 4b,c’). Although AUTEN-99 repressed the activity of MTMR14/hJumpy phosphatase (Fig. 1b), we cannot exclude the possibility that it also affects other phosphatases. Nevertheless, the compound promoted autophagy at the level or upstream of PtdIns3K (Fig. 4b,e’). AUTEN-99 increased autophagic flux in HeLa cells (Fig. 1c,d’) and isolated neurons (Fig. 7a-a”), and elevated the number of autophagic structures in mice (Fig. 2) and *Drosophila* (Fig. 4a,c’), but not or slightly interfered with endocytosis (another membrane generation-dependent cellular process), apoptosis, and stress-induced signaling systems, including the Torc1 (target of rapamycin kinase complex 1) pathway that is an upstream negative regulator of autophagy (Figs S2,S3,S4). In good accordance with these results, AUTEN-99 promoted the survival of mammalian cells, and protected them from undergoing cell death under condition of oxidative stress (Figs 1e,f and 7b). AUTEN-99 also impeded the progression of neurodegenerative symptoms in *Drosophila* models of PD and HD (Figs 5 and 6). These data suggest that AUTEN-99 serves as a potent drug candidate for preventing and treating various age-dependent neurodegenerative diseases, including the most prevalent ones such as PD and HD (see also refs 34 and 35).

AUTEN (autophagy enhancer) molecules target the myotubularin-like phosphatase MTMR14/Jumpy, which antagonizes the type III PtdIns3K being a core component in the mechanism of autophagy, to activate the process in both central nervous system and periphery^34^,^35^ (and see also in this study). Until now, MTMR14/Jumpy was reported as a muscle-specific component and its life-long deficiency in humans was implicated in centronuclear myopathy (a congenital muscle weakness where cell nuclei are abnormally located in skeletal muscle cells) that might be caused by a late-onset inflammation and metabolic dysfunction^43^,^44^. Previously, we demonstrated the expression of *EDTP*, the *Drosophila* ortholog of mammalian *MTMR14/Jumpy* genes, in the fly brain stem^35^. Human expression data (Expression Atlas) also indicate the presence of MTMR14/hJumpy in the nervous system (http://www.ebi.ac.uk/gxa/home). Indeed, MTMR14/hJumpy accumulated in neurons of human brain samples (Fig. 5b). Hence, it seems to be a reasonable approach to inhibit MTMR14/hJumpy temporarily by adding AUTEN-99 at advanced ages in order to promote basal levels of autophagy in neurons. This may help in preventing the onset or decreasing the severity of diverse neurodegenerative conditions.

Pharmacological activation of autophagy has mainly been achieved at the level of upstream regulators of the process. For example, rapamycin promotes autophagic flux via inhibiting the MTOR (mechanistic kinase target of rapamycin) complex 1, which in turn blocks autophagy. Other recently identified autophagy-inducing drugs such as PP242, Torin-1, WYE-354, Ku-0063794, PI-103 and NVP-BEZ235 also interfere with MTOR by binding to its kinase domain^45^. However, there are MTOR-independent autophagy-stimulating agents. Lithium induces autophagy through blocking inositol monophosphatase. while Verapamil and Loperamide elevate autophagic degradation via lowering intracellular Ca^2 + ^levels (*i.e.* by decreasing the level of cAMP)^45^. Autophagy-inducing drugs mentioned above apparently are not specific to autophagy, rather they influence many other cellular processes such as G protein signaling, translation and ribosome biogenesis. In contrast, AUTEN-99 appears to be a specific modulator of autophagy by interacting with MTMR14/Jumpy (Fig. 4c,e’).

Since the incidence of age-dependent pathologies (*e.g.* cancer, neurodegenerative diseases, tissue atrophy, fibrosis, compromised lipid metabolism, immune deficiency and diabetes) generally occurs at advanced ages and they are often associated with dysregulated autophagy^1^, the identification of AUTEN-99 may also have, beyond its medical importance, social and economic significance. In the developed countries, life expectancy is gradually rising and the elderly face heightened threat of acquiring a certain type of degenerative disease. Thus, maintaining basal levels of autophagy by pharmacological means may delay the incidence of such pathologies, thereby lengthening the period of healthy aging. Indeed, promoting basal levels of autophagy in *Drosophila* and mice significantly increases both tissue functioning and organismal survival^24^,^27^,^46^. Furthermore, enhancing the activity of autophagy by a small molecule (AUTEN-67) treatment can extend life span in *Drosophila* even when animals express a toxic, aggregation-prone mutant protein^34^,^35^. As aging can be considered as a collection of seemingly independent degenerative diseases^47^, the effective treatment of such a pathology would only increase the chance of an individual to acquire another type of age-dependent disorder. AUTEN-99 has a general cell protective effect (Figs 1e,f and 7b), and can attenuate the severity of several neurodegenerative conditions (Figs 5 and 6). Based on data we obtained in this study we speculate that administering AUTEN-99 to humans has the potential to improve tissue quality and function, and to extend the period of healthy life span.

## Materials and Methods

### Phosphatase assays

MTMR14/Jumpy protein was purchased from OriGene Technologies (Rockville, USA). The biochemical assay consisted of purified MTMR14 (OriGene Technologies, TP300809) (37 ng/reaction), 100 μM phosphatidylinositol 3-phosphate (Echelon Biosciences, P-3008) in 25 mM Tris (pH = 6) buffer (Molar Chemicals, 09350-101-190) containing 2 mM DTT (Sigma, 17-1318-01) in 10 μl total volume. Reactions were incubated at room temperature, and after 3 hours free phosphate was measured with the Malachite green reagent (Echelon Biosciences, K-1500).

### Assaying autophagy flux in HeLa cells

HeLa cells transgenic for a functional GFP-RFP-LC3B reporter were cultured in DMEM (Dulbecco’s Modified Eagle’s Medium; Sigma, D7777) containing 4500 mg/l glucose, 10% heat inactivated FCS (Life technologies, 10106-169), 40 μg/ml gentamycin (Hungaropharma) and 600 μg/ml G418 (Sigma, G8168). 3 × 104 cells were plated onto 13 mm poly-D-lysine (Sigma, P7405) coated coverslips in 24-well plates (Greiner, 662160) with 24 hours before the treatment. Cells were exposed to different concentrations of drug for 6 hours. As controls, 1% DMSO (Sigma, 41640), 200 nM rapamycin (autophagy inductor) (Sigma, R0395) and 100 nM Bafilomycin A1 (autophagy inhibitor) (Sigma, B1793) were used. For fluorescence microscopy study, cells were fixed in 4% paraformaldehyde (Taab, P001) and mounted in Mowiol 4.88 (Polysciences, 17951-1) supplemented with bis-benzimide (Sigma, B2883) for nuclear staining. 5 epifluorescence pictures were taken in each condition by a BX51 microscope (Olympus, Unicam, Budapest, Hungary) equipped with a FluoViewII camera and the AnalysisPro software (Olympus), using a 60 × /1.4 oil Plan Apochromat objective and the appropriate filter sets (DAPI: BP330-385/DM400/BA420; GFP: BP460-500/DM505/BP510-560; RFP: BP480-550/DM570/BA590). RFP intensity shows soluble and activated LC3B molecules along the whole autophagy process. GFP intensity shows soluble and activated LC3B molecules only in the early stages of autophagy. To study autophagic activity, the number of RFP- and GFP-specific foci was measured in each cell by ImageJ software. Data were analyzed with paired Student’s *t* test, statistical significance was set at p < 0.05.

### Cell viability assays

HeLa cells were seeded into (12-wells) cell culture plates at 105 cells/well density in DMEM (Sigma, D7777) supplemented with 10% FCS (Life Technologies, 10106-169). Cells were treated with AUTEN-99 (at different concentrations) according to the scientific design for 24 hours. Cell viability was measured by the MTT method^47^. Briefly, cells grown in 96-well plates were treated with 3-(4,5-dimethylthiazol-2-yl)-2,5-diphenyltetrazolium bromide (MTT, Sigma, M2128) in a final concentration of 250 μg/ml. After 2 hours of incubation, cells and formazan crystals were dissolved in acidic (0.08 M HCl) isopropanol (Merck, 109634). Optical density was determined at a measuring wavelength of 570 nm against 630 nm as reference with Multiscan EX ELISA reader (Thermo, Bio-science, Budapest, Hungary). Assays were carried out on 6 parallel wells. At least 5 independent viability assays were carried out.

### Culture and drug treatment of flies

The following stocks were obtained from Bloomington Drosophila Stock Center: *ApplGal4* (#32040), *Ddc-Gal4* (#7009), *UAS-myrGFP* (#32199), *UAS-HTT.16Q/CyO* (#33810), *UAS-HTT.128Q* (#33808), *w*^*1118*^ (#5905), *UAS-Hsap\SNCA.A53T* (#8148), *Mi{MIC}EDTP*^*MI08496*^(#44782), *Df(2 R)BSC161* (#9596), *P{EPgy2}EDTP*^*EY22967*^(#22600), *cgGal4* (#7011), *UAS-GFP-2xFYVE* (#42712). *UAS-Parkin-R275W/TM3* was kindly provided by Ng Chee Hoe (NNI, Singapore)^41^, *UAS-mCherry-Atg8a*^48^, *hsFlp; pAct < CD2 < Gal4,UAS-nlsGFP, r4-mCherry-Atg8a*^40^; *hsFlp; r4-mCherry-Atg18a; pAct < CD2 < Gal4,UAS-nlsGFP*, and outcrossed *Syx17*^*LL06330*^ mutants^40^ were gifts from Gabor Juhasz (Eötvös University, Budapest, Hungary). Fly stocks were raised on standard cornmeal-sugar agar medium at 18–25 °C. L3 feeding larvae were treated for 3 hours prior to dissections. Animals were placed into a suspension consisting of instant yeast medium, supplemented by AUTEN-99 solved in DMSO (Sigma, D8418) or the same volume of DMSO only for untreated samples. For analyzing adult animals, flies were placed into vials containing treated medium immediately after eclosion and kept at 29 °C during the entire experiment. AUTEN-99 dissolved in DMSO was added to yeast suspension (final concentration was 100 or 200 μM), and dropped 65 μl to the surface of each vials. Flies were transferred into a fresh vial in every second day.

### Measurement of autophagic structures in the brain of *Drosophila* adults

Flies were maintained on standard cornmeal-sugar agar medium. Experiments were carried out at 29 °C, otherwise indicated. Flies were treated with AUTEN-99 (100 μM, dissolved in DMSO) immediately at posteclosion. Brain from the head of 7 day-old adults was dissected. Images were captured with Zeiss Axioimager Z1 upright microscope (with objective Plan-NeoFluar 20 × 0.3 NA) equipped with an ApoTome, and AxioVision 4.82 and ImageJ 1.45 s software were used to examine and evaluate data. Genotype: w*; UAs-mCherry-Atg8a/UAS-myrGFP; Ddc-Gal4/+.

### Dissection and microscopy of *Drosophila* larval fat body samples

Preparation of fat bodies was carried out in PBS (Sigma, P4417) solution. Covering was achieved in glycerol:PBS (8:2) solution containing Hoechst 33342 (Life Technologies, H-1399) at 10 μM concentration. Microscopy was performed with Zeiss AxioImager Z1 epifluorescence microscope equipped with an ApoTome semiconfocal setup with objective Plan-NeoFluar 40 × 0.75 NA. Images were analyzed using the ImageJ 1.45 s software.

### Measuring PtdIns3P levels and autophagy activity in the fat body of *Drosophila* L3F larvae

The total amount of PtdIns3P-positive structures was examined in *cg-Gal4; UAS-GFP-2xFYVE* animals. The autophagic PtdIns3P pool was revealed in flies transgenic for *hsFlp; r4-mCherry-Atg18a; pAct < CD2 < Gal4, UAS-nlsGFP.* Autophagy activity was assayed in a fly strain with genotype of *hsFlp;pAct < CD2 < Gal4, UAS-nlsGFP, r4-mCherry-Atg8a* at the feeding L3 larval stage. 3 hours prior to dissection, 90–94 hour-old larvae were placed into a suspension consisting of instant yeast medium. AUTEN-99 solved in DMSO was added into final concentrations of 100 or 200 μM. Larvae were treated at 25 °C, and compared with non-treated control ones with the same age and genotype. To examine the effects of AUTEN-99 on autophagic activity in EDTP-defective background, we used an *EDTP* mutant allele, *Mi{MIC}EDTP*^*MI08496*^. Both control *w*^*1118*^ and *Mi{MIC}EDTP*^*MI08496*^ flies were crossed with males carrying a large deletion, Df(2 R)BSC161, which overlaps the *EDTP* genomic region. F1 larvae were raised at 29 °C, and 74–78 hours old L3F larvae were treated with AUTEN-99 (200 μM).

### Testing autophagic activity in fat body cells clonally overexpressing *EDTP*

Tests were performed in a strain derived from crossing between *hsFlp;pAct < CD2 < Gal4, UAS-nlsGFP, r4-mCherry-Atg8a and y*^*1*^ *w*^*67c23*^*; P{EPgy2}EDTP*^*EY22967*^. The fat body of F1 offspring at the L3 larval stage (90–94 hours) clonally overexpresses *EDTP* that antagonizes autophagic membrane formation (*EDTP*-overexpressing fat body cells are green). Animals were treated with AUTEN-99 dissolved in DMSO, untreated controls were treated with DMSO only.

### Climbing assay of *Drosophila* expressing a human mutant Huntingtin protein (128Q-hHTT)

20 adult flies that express the transgene under the control of *ApplGal4* driver were anesthetized, and placed in a vertical glass column (length, 25 cm; diameter, 1.5 cm). After 2 hours of recovery period from CO_2_ exposure, flies were gently tapped 5 times to the bottom of the column. The number of flies that reached the line at 21.8 cm height within 20 sec was counted. Three trials of the three parallel measurements were performed in each experiment. Scores represent the mean percentage of flies that reached the top against the total number tested. Results are presented as mean + /−S.D. Genotypes: *ApplGal4/*+; *UAS-HTT.16Q/*+ and *ApplGal4/*+; *UAS-HTT.128Q/*+.

### Climbing assay of *Drosophila* expressing a human mutant Parkin protein (R275W)

10 female adult flies that express the *UAS-Parkin-R275W* transgene under the control of *Ddc–Gal4* driver were anesthetized, and placed in a vertical glass vial (length, 9 cm; diameter, 2.2 cm). After a 45 min recovery period from CO_2_ exposure, flies were gently tapped 5 times to the bottom of the vial. The number of flies that reached the line at 5 cm height within 10 and 20 sec was counted. 3 parallel experiments and 3 trials were executed. Scores represent the mean percentage of flies that reached the top against the total number tested. Results are presented as mean + /−S.D. of the obtained scores. Genotype: *Ddc-Gal4/UAS-Parkin-R275W.*

### Determining the average speed of flies expressing human mutant form of α-synuclein, A53T

10 female adult flies expressing the *UAS-Hsap\SNCA.A53T* transgene under the control of a *Ddc–Gal4* driver were anesthetized, and placed in a vertical glass vial (length, 9 cm; diameter, 2.2 cm). After a 45 min recovery period from CO_2_ exposure, flies were gently tapped to the bottom of the vial. The speed of individuals that reached the line at 5 cm height within 1 min was calculated. 3 parallel experiments and 3 trials were executed. Results are presented as mean + /−S.D. of the average speed (cm/s). Genotypes: *Ddc-Ga4l/*+ as control and *Ddc-Gal4*/*UAS-Hsap\SNCA.A53T*.

### Western blotting

*On mammalian cells.* Western blot samples were obtained by scraping cells from 6-well plates in 100 μl of hot Laemmli buffer. 25 μl samples were run on a 12% SDS-PAGE and blotted onto Immobilon P PVDF membrane (Millipore, IPVH00010). After blocking with 0.5% blocking reagent (Roche, 1096176) in PBS containing 0.1% Tween 20, filters were probed with specific antibodies anti-LC3 (rabbit, 1:1000; Cell Signaling, 2775), anti-SQSTM1/p62 (rabbit, 1:1000; Sigma, P0067) and anti-GAPDH (rabbit, 1:6000; Sigma, G9545). Proteins were visualized using the ECL system (Luminata Crescendo, Millipore, WBLUR0100). Quantification was carried out by using ImageStudio (LI-COR Biosciences), by referring the intensity of LC3B-II bands to the corresponding GAPDH intensity. Data are shown as a percentage of intensity ratios obtained from DMSO-only treated control cultures and compared by Student’s *t* test (p < 0.05). *On* Drosophila *samples.* Fat body samples from well-fed L3 stage *Drosophila* larvae were dissected. Protein samples of neurodegenerative model flies stem from adult heads. Membranes were probed with anti-Ref(2)P/p62 (rabbit, 1:2500)^49^, anti-EDTP (rat, 1:1000)^34^, alpha-Tub84B (mouse, 1:2500, Sigma, T6199), anti-Atg8a (rabbit, 1:2500)^40^, anti-hHTT (1:1000,Viva Bioscience,VB3130), anti-Ubiquitin (mouse, 1:500, Merck, ST1200), anti-rabbit IgG alkaline phosphatase (1:1000, Sigma, A3687), anti-mouse IgG alkaline phosphatase (1:1000, Sigma, A8438) and anti-rat IgG alkaline phosphatase (1:1000, Sigma, A5153), and developed by NBT-BCIP solution (Sigma, 72091).

### Immunohistochemistry on *Drosophila* brain samples

The immunohistochemistry of Ref(2)P/SQSTM1/p62, polyQ and tyrosine hydroxylase (TH) antibodies were performed as described^35^,^50^, The following primary antibodies were used: anti-Ref(P)2/p62 (rabbit, 1:200)^49^, anti-polyQ (1:100, Merck, MAB1574), anti-TH (1:2000, Temecula, 92590). For nuclear staining, Hoechst 33342 (0.1 mg/ml, Molecular Probes) dye was used. The following secondary antibodies were used: anti-Rabbit Alexa Fluor 488 (Life Technologies, A11008) and anti-Mouse Texas Red (Life Technologies, T862) in 1:500 dilution.

### Life span measurement

Adult female flies were selected for life span determination. Drug treatment was performed as described above. Animals were transferred into fresh medium-containing vials every second day. The number of dead animals was counted daily. Measurements were carried out with three parallels. Mean life spans are presented as mean ± SEM. Genotypes: *w*^*1118*^ and *Syx17*^*LL06330*^.

### Histochemical analysis of human brain samples

Paraffin-embedded human brain tissue samples from temporal cortices (or subfields of these areas) of non-demented aged subjects were obtained from the Netherlands Brain Bank (NBB; Project 598/2009), Netherlands Institute for Neuroscience, Amsterdam. The samples were obtained from clinical patients having no central nervous system medication before death and showed no sign of disease. They were collected from donors for or from whom a written informed consent for a brain autopsy, the use of tissue samples and permission for anonymous use of clinical information was obtained. The sections were deparaffinized, rehydrated and used in light microscopic immunohistochemistry. For the demonstration of the presence of myotubularin-related phosphatase MTMR14/Jumpy, a rabbit polyclonal anti-MTMR14 antibody (ab102575; Abcam, Cambridge, UK) was used. For antigen recovery, deparaffinized sections were boiled in 0.01 M citrate-buffer solution (pH 6.0) in a microwave oven for 2 min (set at 900 watts). After blocking the endogenous peroxidase in 0.1 M TBS containing 3% H_2_O_2_ for 10 min at 37 °C, sections were washed for 3–5 min in 0.1 M TBS (pH 7.4) at room temperature (RT). Tissue sections were next permeabilized, and the background binding of antibody was reduced in a blocking solution (0.1 M TBS containing 5% normal goat serum, 1% BSA, 0.05% Triton X-100) for 30 min at 37 °C. Sections were covered with the above solution containing rabbit anti-MTMR14 primary antibody (1:100 final dilution) at 4 °C for overnight. After incubation with the primary antibodies, sections were washed for 4 × 5 min in 0.1 M TBS (pH 7.4) at RT. Negative control experiments were also performed when the primary antibody was omitted. Sections were then treated with biotinylated anti-rabbit IgG secondary antibody (1:200 final dilution; Amersham Biosciences, Little Chalfont, Buckinghamshire, England) in a blocking solution (where Triton X-100 was omitted) for 5 hours at RT. After several washes (4 × 5 min), biotinylated streptavidin-peroxidase tertiary antibody (1:200 final dilution; Amersham) in a blocking solution (without Triton X-100) was applied to the sections overnight at 4 °C. Sections were washed again in 0.1 M TBS (pH 7.4) for 4 × 5 min at RT, and processed for peroxidase enzyme histochemistry using Sigma Fast DAB Tablet (Sigma, St. Louis, MO, USA) according to the manufacturer’s protocol. Sections were washed for 3 × 5 min in 0.1 M TBS (pH 7.4) at RT, washed in distilled water for 1 min, dehydrated in a series of ethanol solutions, covered with DPX mounting medium (Fluka, 30 Buchs, Switzerland), and coverslipped.

### Cell lines and culture conditions

H9c2 rat embryonal cardiac muscle cells (from ATCC, Rockville, MD, USA) were cultured in Dulbecco’s Modified Eagle’s Medium, containing 10% fetal bovine serum, 4 mM L-glutamine (Sigma-Aldrich, Hungary), 100 U/ml penicillin and 100 μg/ml streptomycin. SH-SY5Y human neuroblastoma cells (from ATCC, Rockville, MD, USA) were cultured in Dulbecco’s Modified Eagle’s Medium, containing 10% fetal bovine serum, 100 U/ml penicillin and 100 μg/ml streptomycin. Each cell type was maintained in 100 mm TC dishes (Orange Scientific, Belgium) in an incubator with humidified air at 37 °C and 5% CO_2_.

### Real-time cell electronic sensing (RT-CES) cytoprotection assay

RT-CES 96-well E-plate (Roche, Hungary) was coated with gelatin solution (0.2% in PBS) for 20 min at 37 °C, then gelatin was washed twice with PBS solution. Growth media (50 μl) (respective to cell types) were gently dispensed into each well of the 96-well E-plate for background readings by the RT-CES system prior to addition of 50 μl of cell suspension. Devices containing the cell suspension were kept at room temperature in a tissue culture hood for 30 min prior to insertion into the RT-CES device in the incubator to allow cells to settle. Cell growth was monitored overnight by measurements of electrical impedance every 5 min. Continuous recording of impedance in cells was reflected by cell index value. Cells were pre-treated (30 min before H_2_O_2_ treatment) with AUTEN-99 next day. H_2_O_2_ concentration to elicit cell injury was 500 μM for both H9c2 rat embryonal cardiac muscle cells and SH-SY5Y neuroblastoma cells. Treated and control wells were dynamically monitored over 24 hours by measurements of electrical impedance every 5 min. The raw plate reads for each titration point were normalized relative to the cell index status right before treatment. Each treatment was repeated in at least 3 wells per plate during the experiments.

### Primary neuronal cell cultures

Primary cortical neurons were prepared from 15 day-old mouse embryos. Embryonic cortices were incubated in 0.05% trypsin solution (Gibco, Life Technologies, 15400-054) for 15 min at 37 °C. After a brief centrifugation step, cells were triturated in NeuroBasal media (Gibco, Life Technologies, 21103-049) supplemented with B27 (Gibco, Life Technologies, 17504-044), 0.5 mM Glutamax (Gibco, Life Technologies, 3505–061), 40 μg/ml gentamicin (Hungaropharma) and 2.5 μg/ml amphotericin B (Sigma, A9528), and filtered through a sterile polyester mesh with 42 μm pore size (EmTek Ltd, Hungary). Cells were seeded onto poly-D-lysine (PDL; Sigma, P7405) coated 6 or 96 well plates at 106 or 8 × 104 cells/well densities, respectively. To prevent the division of non-neuronal cells, cultures were treated with 10 μM cytosine-arabinofuranoside (CAR, Sigma, C6645) on the 2nd day after plating. Oxidative stress was induced on the 7th day of cultivation by 50 or 75 μM H_2_O_2_ for 24 hours, diluted directly from a 30% H_2_O_2_ stock solution (Reanal, 12502-0-48-65) kept at 4 °C.

### Blood-brain barrier penetration

Primary mouse (Balb/c) brain endothelial, pericyte, and astroglia cells were isolated and cultured as described earlier^36^,^37^,^51^. To construct the three cell-type blood-brain barrier model, brain endothelial cells were passaged to the upper side of culture inserts (Transwell, polycarbonate membrane, 0.4 μm pore size, 12-well plate format, Corning Costar), pericytes were seeded on the bottom side of the inserts and astroglia were cultured at the bottom of the wells. Inserts were coated with fibronectin and collagen type IV and the cells received endothelial culture medium in both compartments. Permeability tests for AUTEN-99 were performed on the triple co-culture blood-brain barrier model when the transendothelial electrical resistance showed a tight barrier (243.6 Ω × cm^2^). During the permeability assay culture medium was changed to sterile Ringer-Hepes solution containing 0.1% BSA and ITS (insulin-transferrin-sodium selenite media supplement, PanBiotech). AUTEN-99 was solved in DMSO, and diluted in assay buffer at 10 μM final concentration. Experiments were performed in triplicates, the assay lasted for 1 hour, samples were taken at 30 and 60 min. AUTEN-99 penetration was tested in two directions: AB (from blood to brain) and BA (from brain to blood). Concentrations of AUTEN-99 in samples collected from both compartments were determined by HPLC. As a paracellular permeability marker fluorescein was used^36^,^37^,^51^. The apparent permeability coefficients (P_app_) were calculated as described previously^52^. Briefly, cleared volume was calculated from the concentration difference of the tracer in the lower/basal compartment ([C]_*B*_) after 60 min (t) and upper/apical compartments at 0 hour ([C]_*A*_), the volume of the lower/basal compartment (V_*B*_; 1.5 mL) and the surface area available for permeability (A; 1.1 cm^2^) by the following equation (Equation 1):


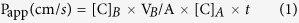


### Quantum chemical calculations

Quantum chemical calculations were performed at the B3LYP/6-31 G(d)//B3LYP/6-31 G(d) level [x01, x02] by the ORCA [x03] quantum chemistry software^53^,^54^,^55^. The partial charges in the optimized geometry were obtained using the CHelpG method^56^,^57^. The atom numbering scheme and results are shown in Fig. 3b.

### Electron Microscopy

Adult male BALB/C mice between 18–20 g b.w. were injected intraperitonially (ip) or added by per os (via the esophagus). AUTEN-99 was dissolved in DMSO and diluted to the requested concentration with physiological saline for the ip injection. Treatments lasted for 60–240 min. Extermination was carried out by cervical dislocation. The administered dose for AUTEN-99 was nearly 390 μM. Controls got physiological saline/DMSO solution. For electron microscopy tissue slices from pancreas, liver and kidney were fixed in cacodylate buffered 2% glutaraldehyde solution. After 1 day fixation the samples were washed, postfixed in 1% osmium tetroxide, embedded in Araldit, and sectioned by Reichert Ultracut microtome. The sections were stained with uranyl acetate and lead citrate, and examined in JEM 1011 electron microscope. All experiments on live vertebrates (mice) were performed in accordance with relevant guidelines and institutional (Eötvös Loránd University, Budapest, Hungary) regulations. All relevant experimental protocols were approved by the relevant local committee in the Institute of Biology at Eötvös University.

### Statistics

Lilliefors test were used to know that the distribution of samples examined is normal or not. If it was normal, F test was performed to compare two variances. If the variances were equal, two-sample Student’s *t*-test was used otherwise *t*-test for unequal variances was applied. If the distribution of a sample is not normal, Mann-Whitney U test was performed^58^,^59^,^60^.

### Ethics Statement

Procedures involving experiments on human subjects (brain samples) are done in accord with the ethical standards of the Committee on Human Experimentation of University of Szeged, Faculty of Medicine and Faculty of Science and Informatics, in which the experiments were done. Procedures involving experimentation on vertebrate animal subjects are done in accord with the guide of Eotvos Lorand University, Budapest, in which the experiments were done.

## Additional Information

**How to cite this article:** Kovács, T. *et al*. The small molecule AUTEN-99 (autophagy enhancer-99) prevents the progression of neurodegenerative symptoms. *Sci. Rep.* **7**, 42014; doi: 10.1038/srep42014 (2017).

**Publisher's note:** Springer Nature remains neutral with regard to jurisdictional claims in published maps and institutional affiliations.

## Supplementary Material

Supplementary Figures S1-S4
