# Supplementary material for: The small molecule AUTEN-99 (autophagy enhancer-99) prevents the progression of neurodegenerative symptoms
Source: Sci Rep. 2017 Feb 16;7:42014. doi: 10.1038/srep42014 (PMC5311965; doi:10.1038/srep42014)
Supplement: Supplementary Figures S1-S4 [file srep42014-s1.doc]

**Supplementary Materials**

**The small molecule AUTEN-99 (autophagy enhancer-99) prevents the progression of neurodegenerative symptoms**

**Tibor Kovács, Viktor Billes, Marcell Komlós, Bernadette Hotzi, Anna Manzéger, Anna Tarnóci, Diána Papp, Fanni Szikszai, Janka Szinyákovics, Ákos Rácz, Béla Noszál, Szilvia Veszelka, Fruzsina R. Walter, Mária A. Deli, Laszlo Hackler Jr., Robert Alfoldi, Orsolya Huzian, Laszlo G. Puskas, Hanna Liliom, Krisztián Tárnok, Katalin Schlett, Adrienn Borsy, Ervin Welker, Attila L. Kovács, Zsolt Pádár, Attila Erdős, Adam Legradi, Annamaria Bjelik, Károly Gulya, Balázs Gulyás & Tibor Vellai**

Supplementary Materials include Suppl. Materials and Methods, Suppl. References, Suppl. Figures S1-S4, and the corresponding figure legends.

**Supplementary Materials and Methods**

**Endocytosis assays.** *In human HaCaT cells:* HaCaT is a spontaneously transformed aneuploid immortal keratinocyte cell line from adult human skin. HaCaT cells transfected with GFP-EGFR, were cultured in Dulbecco Modified Eagle Medium (DMEM) with 1% penicillin-streptomycin and 10% fetal bovine serum. To select GFP-EGFR-transfected cells, the medium was supplemented with 800 μg/ml Geneticin (G418; Sigma, 12061302). After 48 h, fetal bovine serum withdrawal cells were treated with 0, 50, 100, 150 and 200 µM AUTEN-99 in triplicates for 6 h. In the last 30 min, adding 100 ng/ml EGF (epidermal growth factor; Sigma, E9644) induced the expression of GFP-tagged EGF receptor within minutes of exposure to excess ligand. Endocytotic vesicles were detected with confocal microscopy. GFP-tagged EGFR internalization from the cell membrane into the cytosol was examined. *In the fat body of L3 feeding* Drosophila *larvae:* To examine the possible effect of AUTEN-99 on endosome formation, early endosomes were visualized by a Rab5-CFP reporter in the fat body of L3F stage larvae. The Rab5-CFP expressing strain was a kindly gift from Helmut Krämer (University of Texas, Southwestern Medical Center, Dallas, USA). Larvae (86 to 90 h) treated with AUTEN-99 (100 and 200 µM) were compared to well-fed and starved control (untreated) larvae. Starvation was carried out by collecting and putting larvae into 20% (m/V) sucrose-solution for 2 h prior to dissection. The signal of CFP was enhanced by immunostaining of dissected fat bodies. Anti-GFP (1:500; Merck Millipore, MAB3580) and Alexa Fluor 488 anti-mouse IgG (H+L) (1:600; Life Technologies, A-11001) antibodies were used.

**TUNEL assay.** TUNEL- (Terminal deoxynucleotidyl transferase dUTP nick end labeling) assays were performed as described^1^. The following reagents were used: TdT Reaction Equilibrium Buffer (Merck Millpore, S7106), Reaction Buffer (Merck Millpore S7105), TdT enzyme (Merck Millpore, S7107), anti-Digoxigenin-AP (Roche, 11093274910), and NBT/Bcip (Sigma, 72091).

**Semi-quantitative PCR**. Reverse transcription (RT) -PCR experiments were performed using standard protocols. Total RNA and cDNA were prepared using Direct-zol RNA MiniPrep (Zymo Research, R2051) and RevertAid First Strand cDNA Synthesis Kit (Thermo Scientific K1621) from RNAi-treated and control L3 larvae, and used as template for PCR reactions with the following primers: *Gapdh* (used as a housekeeping control gene) 5′-AAA AAG CTC CGG GAA AAG G-3′ and 5′-AAT TCC GAT CTT CGA CAT GG-3′), *dad* (a target gene of BMP - Bone Morphogenetic Protein – signaling)^2^: 5′-AGC CGG CTC TCT ACG AGT C-3′ and 5′-CAC AAT CCC ATC CGT GTA GA-3′, *Socs32E* (a target of JAK-STAT - Janus Kinase/Signal Transducer and Activator of Transcription – signaling)^3^: 5′-TCG TCG AGT ATT GCG AAG TG-3′ and 5′- CTG CTC CCA TTG AAA GTG CT-3′, and *Thor/d4E-BP* (a target gene of the Tor1/insulin/IGF-1 (the target of rapamycin kinase complex 1/insulin-like growth factor -1) signaling axis^4^: 5′-GGA ACC CTC TAC TCC ACC AC-3′ and 5′-GTT CCC CTC AGC AAG CAA C-3′.

**Western blot analysis.** The analysis was performed as described.^5^ Briefly, fat body samples from well-fed L3 stage *Drosophila* larvae were dissected. Membranes were probed with the following primary antibodies: anti-Atg13 (Rat, 1:5000)^5^, anti-P-S6k/Phospho-RPS6KB1 (Rabbit 1:1000, Cell Signaling Technology, 9206) and α-Tub84B (mouse, 1:2500, Sigma, T6199). Anti-rabbit IgG alkaline phosphatase (1:1000, Sigma, A3687), anti-mouse IgG alkaline phosphatase (1:1000, Sigma, A8438), and anti-rat IgG alkaline phosphatase (1:1000, Sigma, A5153) were used as secondary antibodies, and developed by NBT-BCIP solution (Sigma, 72091).

**Supplementary References**

1. McCall, K., Baum, J., Cullen, K. & Peterson, J. Visualizing apoptosis. In *Drosophila Cytogenetics Protocols* (ed. Henderson, D.), Humana Press, pp. 431-442 (2004).
2. Tsuneizumi, K., *et al*. Daughters against dpp modulates dpp organizing activity in *Drosophila* wing development. *Nature* **389**, 627-631 (1997).
3. Callus, B. A. & Mathey-Prevot, B. SOCS36E, a novel *Drosophila* SOCS protein, suppresses JAK/STAT and EGF-R signalling in the imaginal wing disc. *Oncogene* **21**, 4812-4821 (2002).
4. Tettweiler, G., Miron, M., Jenkins, M., Sonenberg, N. & Lasko, P. F. Starvation and oxidative stress resistance in *Drosophila* are mediated through the eIF4E-binding protein, d4E-BP. *Genes Dev*. **19**, 1840-1843 (2005).
5. Takáts, S., Varga, Á., Pircs, K. & Juhász, G. Loss of Drosophila Vps16A enhances autophagosome formation through reduced Tor activity. *Autophagy* **11**, 1209–1215 (2015).

**Supplementary Figure S1**

**
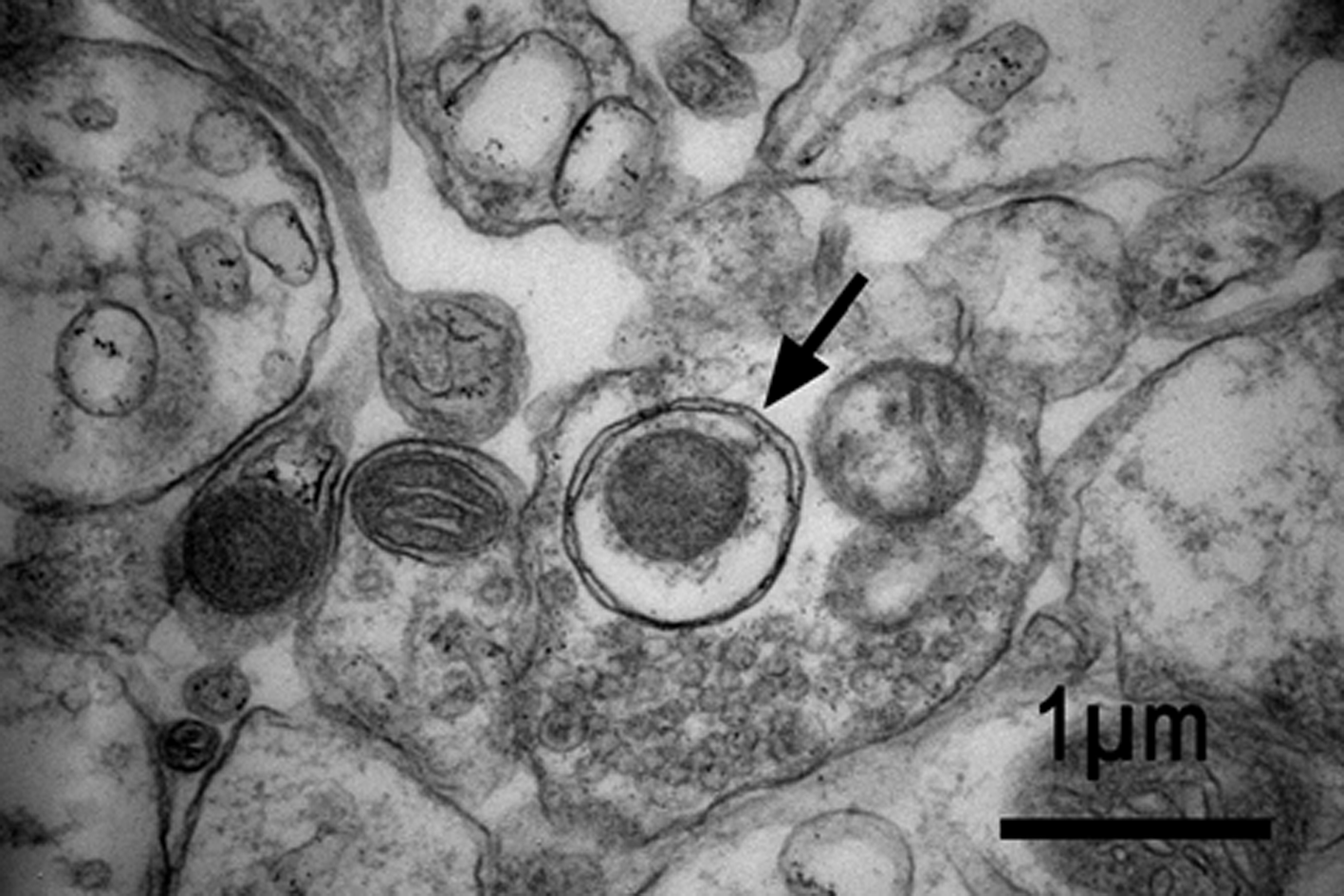
**

**Suppl. Figure S1. Autophagic activity in the brain sample of a mouse treated with AUTEN-99.** TEM (transmission electron microscopy) image showing an autophagosome (indicated by the arrow). Note that in untreated animals the amount of macroautophagic structures in neurons did not reach detectable levels.

**Supplementary Figure S2**

**
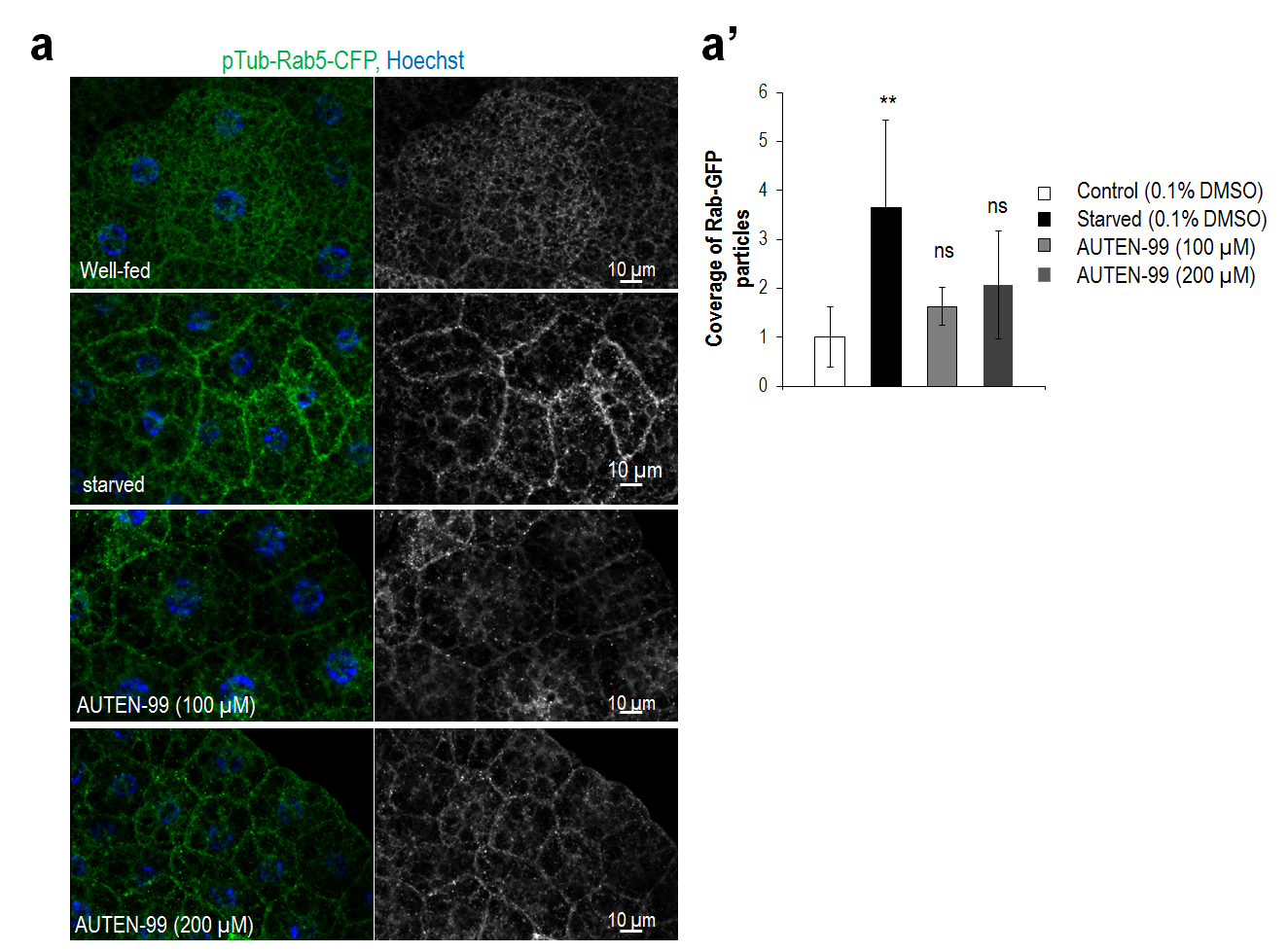
**

**
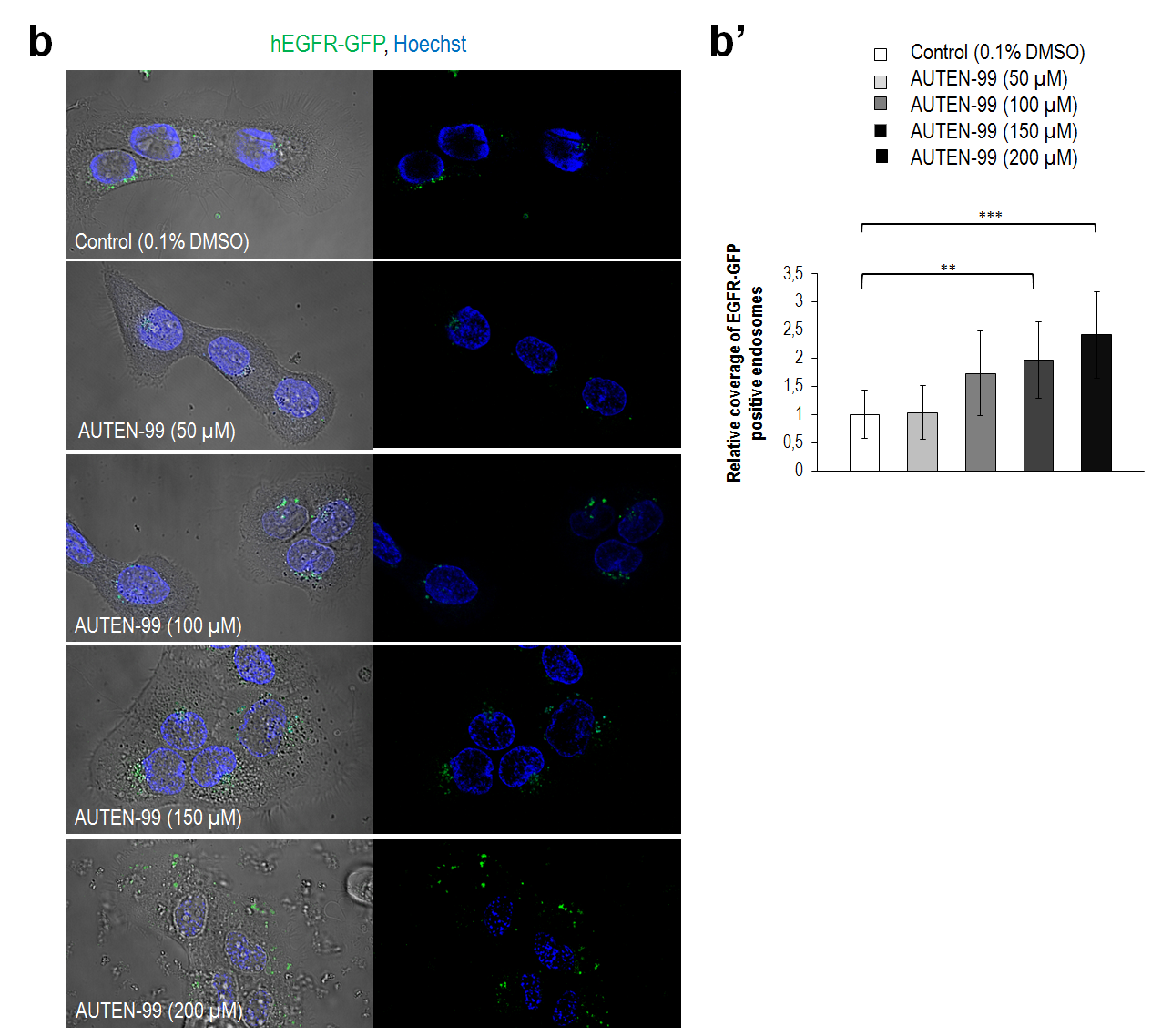
**

**Suppl. Figure S2. AUTEN-99 does not influence endocytosis in *Drosophila* and in human cells**. (**a**) *Drosophila* fat body cells expressing the sorting endosome marker Rab5-CFP (cyan fluorescent protein enhanced by anti-GFP IHC) were treated with AUTEN-99 at different concentrations. Untreated samples serve as control. Colored and the corresponding uncolored fluorescence images are shown side by side. (**a’**) Quantification of sorting endosomal structures in untreated versus AUTEN-99-treated *Drosophila* fat body cells transgenic for a Rab-5-CFP reporter. “ns” denotes not significant. (**b**) AUTEN-99 slightly increases the amount of hEGFR-labeled endocytic structures in human cells. (**b’**) Quantification of EGFR-positive structures in untreated versus AUTEN-99-treated cells. In panels **a** and **b**, Hoechst staining (blue) indicates nuclei. In panels **a’** and **b’**, bars represent mean ±S.D., *: P<0.05; **: P<0.01; ***: P<0.001; unpaired Student’s *t*-test.

**Supplementary Figure S3**

**
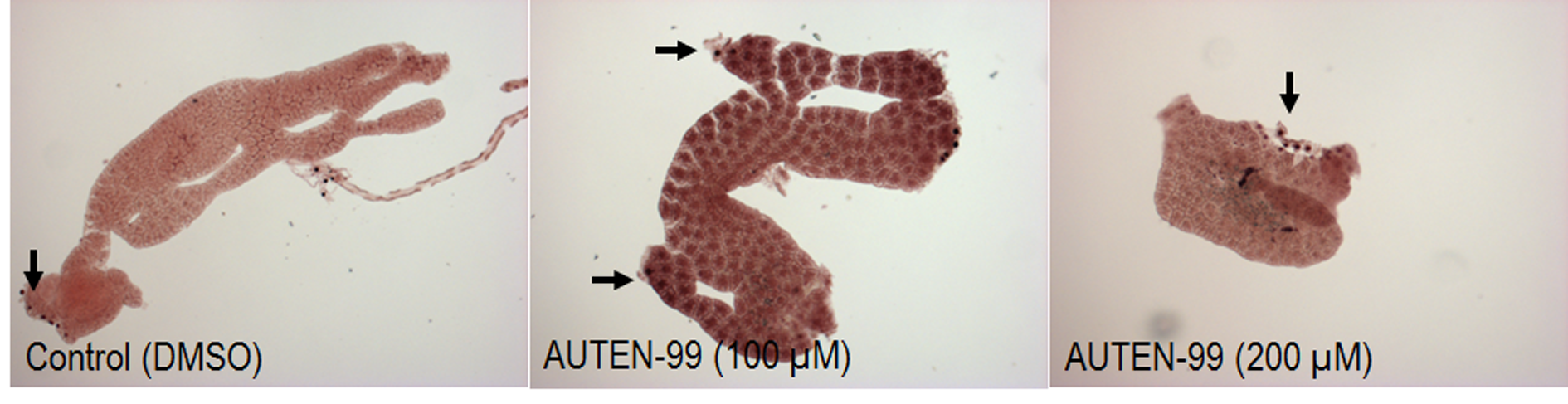
**

**Suppl. Figure S3. AUTEN-99 does not trigger apoptosis.** Fat body from L3F stage *Drosophila* larvae was assayed by TUNEL staining. Arrows indicate TUNEL-positive cells that undergo apoptosis. This cell death results from mechanical damage caused by handling with tweezer. Other parts of the samples examined do not display positive staining in both control (left panel) and treated animals (middle and right panels).

**Supplementary Figure S4**


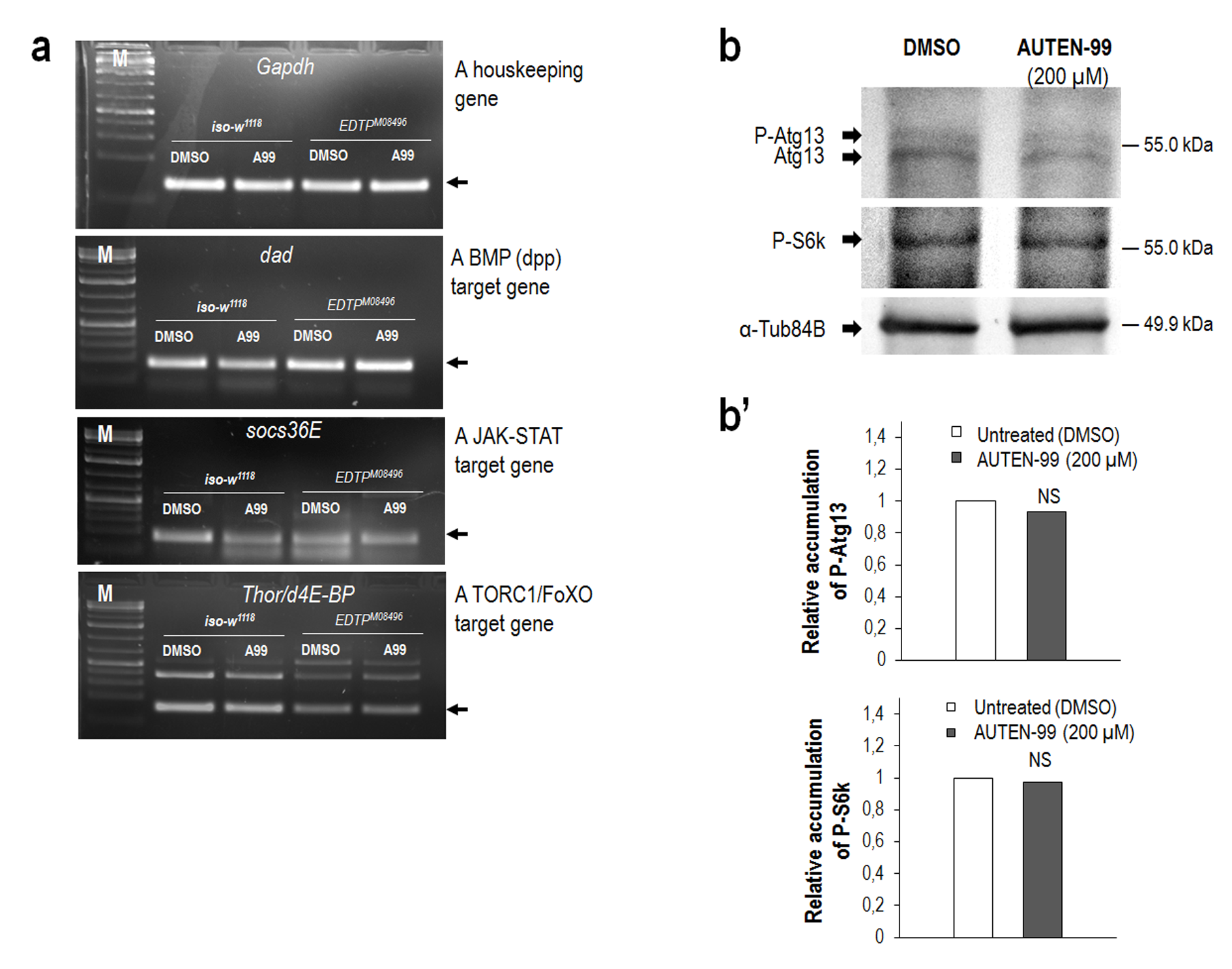


**Suppl. Figure S4. AUTEN-99 does not interfere with signaling systems involved in cellular stress response.** (**a**) Semi-quantitative PCR experiments demonstrating relative transcriptional activity of target genes of signaling systems examined. AUTEN-99 treatment does not lead to changes in the transcription of genes assayed. The dTor1 complex (TORC1) inhibits *d4E-BP* transcription via interfering with dFoXO, the target of insulin/IGF-1 signaling. Abbreviations: M, molecular weight marker; *iso-w^1118^*, (control) wild-type animals; *EDTP^M08496^*, EDTP deficient animals; A99, AUTEN-99; Gapdh, Glyceraldehyde-3-phosphate dehydrogenase; dad, daughters against dpp; dpp, decapentaplegic; BMP, bone morphogenetic protein; socs36E, the suppressor of cytokine signaling; JAK-STAT, Janus Kinase and Signal Transducer and Activator of Transcription; 4E-BP, Eukaryotic translation initiation factor 4E-binding protein; Tor1, Target of Rapamycin kinase complex 1. Arrows indicate the relevant bands. (**b**) Western blot analysis shows that AUTEN-99 does not interfere with the Tor complex 1 (Torc1), an upstream negative regulator of autophagy. Torc1 influences the phosphorylated level of S6k/RPS6KB1 and Atg13 proteins (the latter is a part of the autophagy-activating Atg1 kinase complex), and AUTEN-99 treatment does not modify P-Atg13 and P-S6k levels. α-Tub48B served as an internal control. DMSO, untreated control. (**b’**) Quantification of relative P-Atg13 and P-S6k levels shown on the blot in panel **b**. NS, not significant.
